# The role of iron in pulmonary pathology

**DOI:** 10.1186/s40248-015-0031-2

**Published:** 2015-12-01

**Authors:** Heena Khiroya, Alice M. Turner

**Affiliations:** School of Clinical and Experimental Medicine, University of Birmingham, Birmingham, B15 2TT UK; Centre for Translational Inflammation Research, Queen Elizabeth Hospital, Mindelsohn Way, Edgbaston, Birmingham, B15 2GW UK; Heartlands Hospital, Bordesley Green East, Birmingham, B9 5SS UK

**Keywords:** ARDS, Cancer, Cigarette, COPD, Infection, IREB2, Iron

## Abstract

Respiratory disease accounts for a large proportion of emergency admissions to hospital and diseaseassociated mortality. Genetic association studies demonstrate a link between iron metabolism and pulmonary disease phenotypes. *IREB2* is a gene that produces iron regulatory protein 2 (IRP2), which has a key role in iron homeostasis. This review addresses pathways involved in iron metabolism, particularly focusing on the role of *IREB2*. In addition to this, environmental factors also influence phenotypic variation in respiratory disease, for example inhaled iron from cigarette smoke is deposited in the lung and causes tissue damage by altering iron homeostasis. The effects of cigarette smoke are detailed in this article, particularly in relation to lung conditions that favour the upper lobes, such as emphysema and lung cancer. Clinical applications of iron homeostasis are also discussed in this review, especially looking at the pathophysiology of chronic obstructive pulmonary disease, lung cancer, pulmonary infections and acute respiratory distress syndrome. Promising new treatments involving iron are also covered.

## Introduction

Environmental risk factors, such as smoking status and air pollution, interact with genes, in order to produce pathology. The variation of phenotypes seen in patients with the same diagnosis can be vast, even if they have been exposed to the same environmental factors. These differences in chronic disease manifestations point towards a clear genetic component. Understanding the interaction between the environment and genetics can theoretically allow us to predict susceptibility towards certain diseases and target treatments. Respiratory disease is a great socioeconomic burden on the UK’s health services, with it accounting for 1 in 5 deaths, and being the second most common cause of emergency admissions [1]. It is therefore important to understand what drives pulmonary pathology.

The lungs are continually exposed to metals in the atmosphere. Iron is in great abundance in the earth's core [2] and from here is able to dissolute into the atmosphere. Inhalational iron therefore may be a source of environmental variation within respiratory disease. Iron is also found in cigarette smoke, the environmental factor with the strongest causative link to pulmonary pathology. Through cigarette smoking, iron has been shown to alter disrupt homeostasis in the lung, making the tissue more susceptible to damage [3]. Iron concentrations in lung cell lines and bronchoalveolar lavage (BAL) fluid have been studied, and are increased in cases of disease [4].

Alterations in iron homeostasis have also been examined from a genetic viewpoint. Iron responsive element binding protein 2 (*IREB2*) is a gene on chromosome 15, and its protein product is iron regulatory protein 2 (IRP2): a key player in maintaining iron balance. *IREB2* is in strong linkage disequilibrium (LD) with nicotine receptor genes (*CHRNA3* and *5*) [5] and it is this that led *IREB2* to be investigated in relation to respiratory conditions such as chronic obstructive pulmonary disease and lung cancer. Interest in iron came later as a result of *IREB2* genetic association studies to establish links between iron and pulmonary phenotypes. This article reviews the role of iron metabolism and iron homeostasis in lung disease, particularly focusing on *IREB2*.

## Review

### Cellular iron balance

Iron is most commonly found in ferric (Fe^3+^) and ferrous (Fe^2+^) states; the more stable of these under normoxic conditions is Fe^3+^. Fe^2+^ reduces oxygen to form superoxide radicals [6] which cause damage to cells and ultimately result in apoptosis [7]. To reduce the toxicity of free iron, homeostatic mechanisms are in place to ensure appropriate systemic and intracellular iron conditions. Iron is stored in hepatocytes, and macrophages in the liver and spleen. These levels remain constant in spite of fluctuations in the diet. The main regulator of systemic iron control is hepcidin [8], a hormone produced in the liver. Dietary iron is absorbed across the duodenal mucosa; modulation of this is controlled by signals from the liver [8, 9]. Increasing levels of iron in the plasma induce HAMP, a protein that encodes hepcidin production. Hepcidin then reduces the amount of iron moving from stores into the plasma as well as the amount absorbed from the diet. Conversely, when plasma levels of iron are low, hepcidin production is downregulated in order to increase the amount of iron that can be moved from hepatocytes and macrophages, as well as absorption from dietary sources [10].

**Fig. 1:**
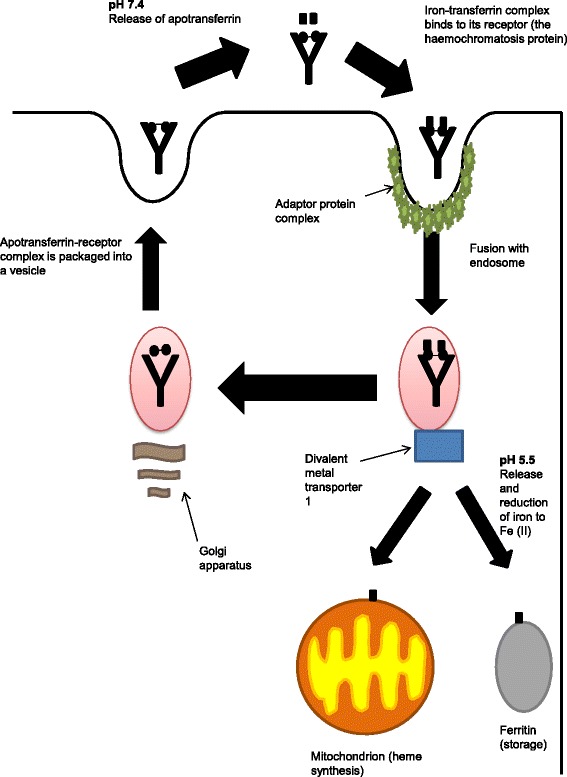
Iron homeostasis: the transferrin-to-cell cycle. At neutral pH, apotranferrin is released at the cell membrane. The iron-transferrin complex then binds to its receptor at the cell membrane and endosomal fusion occurs. A complex is then formed with divalent metal transporter 1. At pH 5.5 iron is reduced and released to be used by the mitcochodria or stored as ferritin. The Golgi body package the apotransferrin-receptor complex into a vesicle and it is transported back to the cell membrane

There is a separate second system that regulates iron homeostasis within cells. In the bloodstream, iron binds to transferrin [11]. Transferrin receptor 1 is expressed on the cell membrane, and most cells can regulate the influx of iron via this channel [12]. Figure 1 demonstrates the transferrin-to-cell cycle in more detail. Non-tranferrin bound iron species are found in the plasma, the main form is thought to be Fe^3+^ bound to citrate [13]. Mechanisms surrounding cellular uptake are unknown but thought to be independent of endocytosis [13]. Iron regulatory proteins 1 and 2 (IRP1 and IRP2) register iron concentrations in the cytosol [14] and post-transcriptionally regulate expression of transferrin receptors and iron metabolism genes to optimise cellular iron availability [15]. Macrophages provide an additional route for iron concentrations to be maintained intracellularly via phagoytosis of damaged erythrocytes [10]. The phagocytosed iron is either stored as ferritin in the cytoplasm and is subject to regulation by the IRPs, or travels through ferroportin to the extracellular fluid [10].

Genetic ablation studies in mice have shown that IRP2 has a key role in mammalian iron metabolism [16, 17]. In contrast, the absence of IRP1 has little effect on iron homeostasis because IRP2 is able to increase its activity in a compensatory fashion [18]. Under conditions of iron starvation, IRPs bind to specific RNA stem-loop structures called iron responsive elements (IREs) to stabilise the transferrin receptor and inhibit the translation of ferritin mRNA [15]. Iron dissociates from transferrin, and ferritin biosynthesis is prevented, therefore iron is not stored and able to be metabolised [19]. When cells are abundant with iron; sulphur clusters with iron at the core of IRP1 [15], whereas IRP2 undergoes proteosomal degradation [20]. The result of both these actions is inactivation of the IRPs, which causes transferrin receptor 1 mRNA degradation and ferritin mRNA translation. This ensures less iron is taken into cells via transferrin receptor 1, and free iron is stored in the form of ferritin [19].

Unlike IRP1, IRP2 remains stable under hypoxic conditions and is highly active with regards to binding IREs [15]. Hypoxia-inducible factors activate the IRP genes to increase the amounts of iron available for erythropoiesis [21]. Hypoxia appears to increase IRP2 levels by a post-translational mechanism involving protein stability [22]. Ischaemia-reperfusion injury of the lung following prolonged hypoxia shows elevated levels of iron [23], which is important to note, as both type 1 and type 2 respiratory failure are characterised by hypoxia.

### The effects of cigarette smoke

Particulate matter (including iron) from cigarette smoke is deposited in the lungs and can cause damage by altering iron homeostasis. Lung cells are different from other cell lines in that they are continually exposed to changes in oxygen levels, which also have effects on iron homeostasis. A study done in rats showed that airway iron was elevated after exposure to cigarette smoke, which led to increased oxidative stress and release of IL-8 [3]. Generation of oxidative stress and cytokine accumulation contribute to tissue damage by the process of neutrophil recruitment [24].

Conditions such as emphysema and lung cancer, which have a predilection for the upper lobes of the lungs, may be due to regional variation of iron. In one study, BAL was taken from the upper and lower lobes of the lungs of smokers and nonsmokers. Fluid from the upper lobes of those who smoked had significantly higher concentrations of extracellular ferritin-bound iron and less transferrin, which may contribute to the pathogenesis of emphysema and lung cancer via oxidative stress [25]. Another study demonstrated that excess iron in the lung influenced the production of human alveolar macrophage-derived IL-1beta, and this also showed regional variation [26], giving further support to the link between iron and inflammation, and clinical relevance to lung conditions that favour the upper lobes.

Maternal smokers have been shown to have a higher ferritin level in their placental cord blood than non-smoking women, however there was a negative correlation between maternal smoking and infants’ ferritin and total body iron [27].

Iron in the lung has been shown to increase with age, and again with smoking, although this effect is separate from senescence [28]. This increase in iron could account for the increased risk of lung injury seen with age [29]. Areas of emphysema in comparison to normal lung tissue have shown greater uptake of the polyclonal antibody for ferritin, and the increase in staining was not limited to alveolar macrophages but was seen throughout the epithelium [28]. This suggests that quick responses to iron are seen throughout the lung; with cells trying to limit oxidative damage by storing iron as ferritin.

### Chronic Obstructive Pulmonary Disease (COPD)

*IREB2* has been implicated as a COPD susceptibility gene in a case–control study [30]. Expression of IRP2 was higher in the lung tissue samples of those with COPD, and there was a trend for association for five *IREB2* single-nucleotide polymorphisms (SNPs) with upper lobe emphysema [30]. Table 1 demonstrates combined *p* values of the seven SNPs investigated in this study. Three of the SNPs investigated in this study were replicated in a later study [31]. The role of *IREB2* and iron homeostasis was felt to be independent of the effect on lung function as there was no association between FEV_1_ and these SNPs [31]. Another paper showed that after adjusting for *CHRNA3* and *CHRNA5* (genes that are in strong LD with *IREB2* and also known to be associated with COPD) this association was no longer significant [5]. Tight LD on this area of chromosome 15 complicates isolation of causal genes for COPD. The possibility that there are multiple functional genes in this locus controlling for different aspects of COPD is a valid one; *CHRNA3* and *CHRNA5* have been shown to be significantly associated with pack/years smoking and emphysema in COPD patients, whereas the most significant association for FEV_1_ was in an intron of *IREB2* [32]. Further support for this is shown in a paper that demonstrates the effect of *IREB2* on COPD is independent from smoking [33]. Long-range control of gene expression is another concept which should be considered, with greater than 85 % of variants being associated with disease traits outside the coding region of annotated genes [34].

**Table 1:** *IREB2* single nucleotide polymorphisms and their association with chronic obstructive pulmonary disease

SNP	Risk allele	Minor allele	Combined *p* using Fisher’s exact method
rs2568494	A	A	1.64 × 10^−7^
rs2656069	T	C	1.03 × 10^−5^
rs1964678	G	A	5.94 × 10^−4^
rs12593229	G	T	9.21 × 10^−4^
rs10851906	A	G	1.65 × 10^−5^
rs965604	A	G	5.42 × 10^−4^
rs13180	T	C	6.42 × 10^−4^

Alpha-1 antitrypsin deficiency (AATD) is a genetic disorder that may manifest with symptoms of COPD [35]. Severe AATD accounts for 0.63 % of usual COPD [36]. In UK National Registry of AATD, *IREB2* SNP rs2568494 was shown to be significantly associated with emphysema, and this effect appeared to be more prominent in males [37]. This cohort of patients also showed significant SNP-by-smoking interactions, contrasting the research done in usual COPD [32]. Severity of AATD patients has been shown to correlate with iron, with patients with ZZ phenotype having significantly more ferritin and non-heme iron than those with MM phenotype [38].

Neutrophil elastase and oxidative stress is increased in the typical AATD lung. When neutrophil elastase levels are raised, levels of iron in the airways also increase when bronchoalveolar fluid is examined, but ferritin is degraded: this could be to increase the overall extracellular iron pool for cellular uptake [39].

### Lung cancer

As with COPD, promising candidate genes such as *CHRNA3* and *CHRNA5* have been discovered in association with lung cancer [40], and as discussed in the section on COPD, these genes are in strong linkage disequilibrium with *IREB2*. As well as being identified in relation to lung cancer, these genes are also relevant to nicotine addiction [41–43]. It is interesting that both COPD and lung cancer are pathologies that are strongly linked to cigarette smoking, dependence on which could be linked to the same SNPs. The same genetic and environmental risk factors are shared for these diseases, meaning presence of these SNPs could confer increased susceptibility for all three things.

IRP2 has pro-oncogenic activity in human lung cancer cells, and this is variable depending on a specific 73 amino acid insert [44]. This pro-oncogenic activity is not able to proceed without IRP2: a causal relationship has been established by turning off the expression of *IREB2* [44]. Depleting cancer cells of iron has been hypothesised as a potential therapy. Promising new research is emerging on the use of iron-chelating agents to treat lung cancer [45].

### Susceptibility to lung infections

During times of infection, iron homeostasis adjusts so that cytokines scavenge iron from tissues and sequester it within macrophages. Microbes require iron to proliferate, and so the purpose of this is partly to deny invading organisms the iron they need; and partly to protect the host from the toxic effects of Fe^2+^ and the subsequent formation of superoxide radicals via the Fenton reaction that may be released during inflammation [10]. Microbes can produce small compounds called siderophores, which scavenge iron from the host by forming complexes which are then taken up by active transport [46]. In response, the host tries to make less iron available for the microbe, which is a state known as “the anaemia of inflammation” [4]. The iron status of the host is very important in determining risk of pulmonary infection. For example, correction of iron deficiency led to activation of previously suppressed pre-existing infections including malaria, brucellosis and tuberculosis, in a group of Somali nomads [47]. As iron repletion advanced, infectious activity reached a peak, showing that iron repletion can allow infectious diseases to become more clinically overt [47].

*Mycobacterium tuberculosis* employs the siderophore system to acquire iron [48]. The link between iron and tuberculosis virulence is further confirmed by studies into dietary iron. By increasing dietary intake of iron the host macrophages appear to be overloaded by iron and unable to suppress the spread of pulmonary tuberculosis [49]. *In vitro *work has shown that by deleting the gene that codes for siderophore production in *Myocbacterium tuberculosis*, it is unable to grow and replicate within macrophages [48]. This suggests that siderophore biosynthesis may be a promising candidate for new antibiotics to target with regards to tuberculosis treatment. Indeed, a siderophore analog has been coupled with the antimalarial artemisinin, and has successfully been shown to retain antimalarial properties as well as combat tuberculosis *in vitro *[50]. More recently a further siderophore-independent pathway has been established, where *Mycobacterium tuberculosis* is able to use free heme and heme from haemoglobin as an iron source [51], providing a new line of investigation for future therapies to target.

Patients with severe pneumonia have been shown to have elevated levels of sideromacrophages in their BAL fluid [52], reflecting higher levels of iron in the airways of these patients. Animal models have also shown that iron acquisition is critically important in the pathogenesis of *Staphylococcus aureus* pneumonia [53] and *Klebsiella pneumoniae* pneumonia [54]. Host defences, such as production of the protein lipocalin 2, are able to bind the main siderophores produced by *Klebsiella pneumoniae* and stop them from scavenging iron [54]. *Streptococcus pneumoniae* growth has been observed in media containing differing levels of iron and manganese. When the iron content was high iron homeostasis was disrupted and the bacteria were susceptible to oxidative stress [55]. However, manganese proved to protect the bacteria via its antioxidant effect and competed with iron for transport into the bacterium [55]. This work demonstrates the role of other transition metal ions with regards to pulmonary inflammation. The mechanisms by which lipocalin 2 protects the host, and manganese works to protect the bacteria, could be exploited with regards to treatment of pneumonia. To our knowledge, there is a lack of published work on therapies targeting iron in pneumonia.

Gram negative lung pathogens such as *Haemophilus influenza* have developed strategies to acquire iron by using iron-containing human proteins such as tranferrin, lactoferrin, haemoglobin and ferritin [56–58]. The bacterial receptors have evolved to compete with human receptors and transferring binds to bacterial receptors preferentially [59]. Gram positive species such as *Streptococcus pneumoniae* have also developed iron-acquistion strategies separate from siderophores. Hemophores are proteins that bacteria secrete to bind heme from the host [60]. As well as indirectly using heme chelation, *Streptococcus*, like the Gram negative bacteria, can directly acquire iron by hijacking iron-containing human proteins [60].

Another pulmonary pathology that demonstrates that iron is key for airway bacterial growth is cystic fibrosis (CF). Elevated levels of iron have been found in the respiratory tracts of these patients, implying disrupted iron homeostasis [61]. *Pseudomonas aeruginosa* is the micro-organism responsible for the majority of CF infectious exacerbations. Around 6 % of the genes in *Pseudomonas aeruginosa* are iron-responsive [62], and they take some responsibility for production of the siderophores that allow microbes to scavenge iron from the host. *Pseudomonas aeruginosa* exists in biofilms, which make it difficult for antibiotics to eradicate this microbe [61]. Iron also plays a role in strengthening the structure of these biofilms [63], enabling the bacteria to have an extra defence against the host.

*Pseudomonas aeruginosa* may also acquire iron from the host via siderophore-independent pathways. For example, proteases employed by microbes have been shown to cleave iron from host proteins in order to utilise the iron for their own purposes [64]. Pulmonary haemorrhage is a common complication of CF exacerbations. During these episodes heme-bound iron can be taken up via two separate pathways, but no formal data exists on these as yet [61].

Novel adjuvants to anti-pseudomonal antibiotics are under investigation, namely iron chelators such as gallium and desferrioxamine [65]. *In vitro *work has shown that a formulation containing gentamicin and gallium is more effective than gentamicin alone against *Pseudomonas aeruginosa* grown within a biofilm [66]. Desferrioxamine in combination with tobramycin was efficacious at disrupting established biofilms, as well as preventing the formation of new biofilms on epithelial cells from CF patients [67].

### Acute Respiratory Distress Syndrome (ARDS)

ARDS is a severe form of inflammatory lung disease. Ferritin levels have been shown to be a marker of both developing ARDS and multiple organ failure in trauma patients [68]. The authors of this study discuss that ferritin could be raised as a result of being induced by proinflammatory cytokines, however the role of ferritin as an acute phase reactant is not discussed as a confounder in the paper. Ferritin was not associated with other markers of clinical injury in this study, for example, PaO_2_/FiO_2_ ratio, days requiring ventilation, and mortality [68]. Samples of BAL fluid from those with ARDS have significantly higher levels of iron than samples from healthy volunteers [69]. This lends support to the hypothesis that disrupted iron homeostasis causes increased oxidative stress and tissue damage in people with ARDS.

Transfusion-related acute lung injury (TRALI) is a form of ARDS associated with the process of blood transfusion. Pathogenesis of TRALI is unclear, but it has been suggested that iron in the blood components being transfused elevate iron levels in the recipient, and this causes damage to the cells by disruption of iron homeostasis [70]. At present, there are no clinical or *in vitro *trials aimed at stopping this.

### Recent advancements

Over the last two years there has been increased interest in the role of iron in pulmonary pathology. The majority of research has been looking into how iron oxide particles can be used as novel nanoprobes to diagnose and treat lung disease. Superparamagnetic iron oxide nanoparticles have successfully been administered into lungs and detected on magnetic resonance imaging [71]. In mice, these probes have been used to visualise areas of angiogenesis in lung cancer [72]. *In vitro *drug delivery in lung cancer has been successfully developed by using these nanoparticles coated in gelatin [73].

## Conclusion

The role of iron in pulmonary pathology has been well established, both from an environmental viewpoint as well as in terms of genetic susceptibility. Disruption of cellular iron homeostasis appears to have an adverse effect on the lung. Further work on this metal could look at the role of iron in prevention and treatment of pulmonary pathology, either by dietary deficiency, venesection or chelator therapy.

## Abbreviations

AATD: Alpha-1 antitrypsin deficiency
ARDS: Acute respiratory distress syndrome
BAL: Bronchoalveolar lavage
CF: Cystic fibrosis
CHRNA: Cholinergic receptor, neuronal nicotinic, alpha
COPD: Chronic obstructive pulmonary disease
FEV_1_: Forced expiratory volume in 1 second
IRE: Iron responsive element
IREB: Iron responsive element binding protein
IRP: Iron regulatory protein
LD: Linkage disequilibrium
mRNA: messenger ribonucleic acid
SNP: Single nucleotide polymorphism
